# The effect of whey protein on viral infection and replication of SARS-CoV-2 and pangolin coronavirus in vitro

**DOI:** 10.1038/s41392-020-00408-z

**Published:** 2020-11-24

**Authors:** Huahao Fan, Bixia Hong, Yuqian Luo, Qi Peng, Liqin Wang, Xiangshu Jin, Yangzhen Chen, Yunjia Hu, Yi Shi, Tong Li, Hui Zhuang, Yi-Hua Zhou, Yigang Tong, Kuanhui Xiang

**Affiliations:** 1 Department of Microbiology and Infectious Disease Center, School of Basic Medical Sciences, Peking University Health Science Center, Beijing, 100191 China; 2 Beijing Advanced Innovation Center for Soft Matter Science and Engineering, College of Life Science and Technology, Beijing University of Chemical Technology, Beijing, 100029 China; 3 Department of Laboratory Medicine, Nanjing Drum Tower Hospital, Nanjing University Medical School, Nanjing, 210008 China; 4 CAS Key Laboratory of Pathogenic Microbiology and Immunology, Institute of Microbiology, Chinese Academy of Sciences, Beijing, 100101 China; 5 Department of Infectious Diseases, Nanjing Drum Tower Hospital, Nanjing University Medical School, Nanjing, 210008 China

**Keywords:** Drug development, Infectious diseases

**Dear Editor**,

Since severe acute respiratory syndrome coronavirus 2 (SARS-CoV-2) RNA has been detected in human breastmilk, infants’ safety with breastmilk feeding is of great concern for women with coronavirus disease 2019 (COVID-19).^1^ It is known that milk has antiviral properties.^2^ However, little is known about the antiviral property of human breastmilk to SARS-CoV-2 and its related pangolin coronavirus (GX_P2V). Here we present for the first time that whey protein from human breastmilk effectively inhibited both SARS-CoV-2 and GX_P2V by blocking viral attachment and viral replication at entry and even post entry. Moreover, human whey protein inhibited infectious virus production, as proved by the plaque assay. We found that whey protein from different species, such as cow and goat, also showed anti-coronavirus properties. Commercial bovine formula milk also showed similar anti-SARS-CoV-2 activity.

Firstly, healthy human breastmilk samples collected in 2017 and stored properly at −80 °C were tested for their potential effects on SARS-CoV-2 infection. Mothers provided informed consent. This study was approved by the ethics committees of the Medical Center and all samples were anonymized. The skimmed breastmilk was obtained after removal of the lipid fraction. Vero E6 cells were infected with a mixture of SARS-CoV-2 pseudovirus (650 TCID_50_/well) and human breastmilk (4 mg/ml). Human breastmilk from eight donors showed a significant inhibition of more than 98% of the SARS-CoV-2 pseudovirus. As reported recently, a SARS-CoV-2-related pangolin coronavirus model (GX_P2V)^3^ shares 92.2% amino acid identity in spike protein with SARS-CoV-2, which is a suitable model for SARS-CoV-2 infection research. We utilized GX_P2V (MOI: 0.01 in Vero E6 cells) as the model to study the effect of breastmilk on viral infection and also found similar results (Fig. 1a). The inhibition is concentration dependent with an EC_50_ of 0.13 mg/ml of total protein (Fig. 1b and Supplementary Fig. S1) in the SARS-CoV-2 pseudovirus model. Consistent with the SARS-CoV-2 study, the GX_P2V model also showed inhibition with an EC_50_ of about 0.5 mg/ml of total protein (Fig. 1c and Supplementary Fig. S2). Interestingly, human breastmilk did not show any cytotoxicity to Vero E6 cells (CC_50_ > 3 mg/ml), and even promoted cell proliferation. These results indicated that human breastmilk showed high anti-SARS-CoV-2 and anti-GX_P2V property, but limited cytotoxicity to Vero E6 cells.

**Fig. 1:**
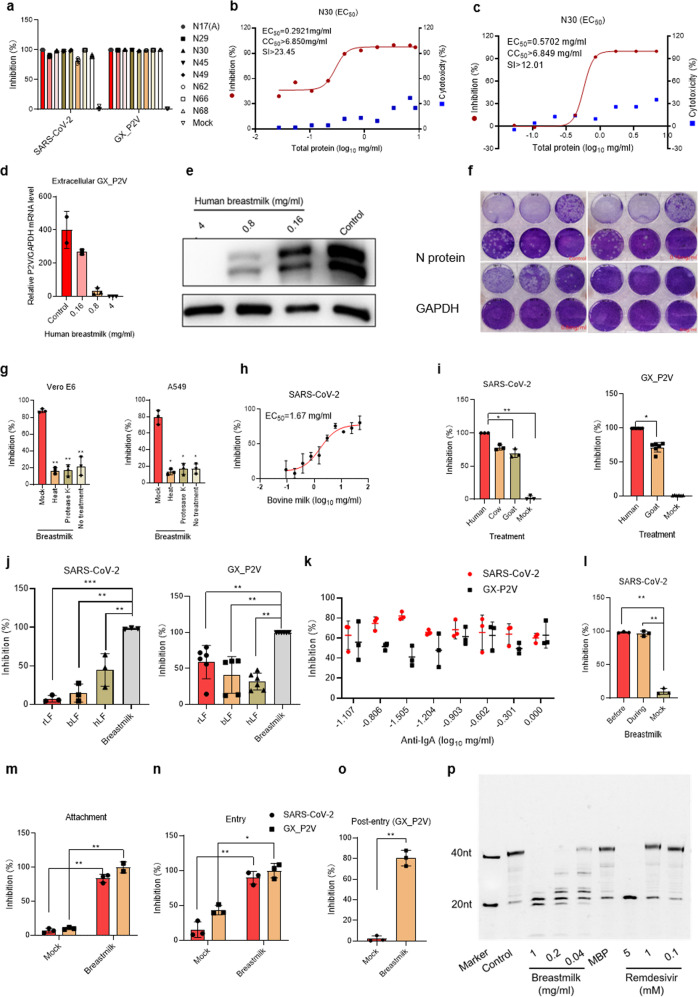
Inhibition of SARS-CoV-2 and GX_P2V by whey protein from breastmilk. **a** Luciferase assay and RT-qPCR analysis of the impact of breastmilk (4 mg/ml) from different donors on SARS-CoV-2 and GX_P2V. **b**, **c** Inhibition analysis of SARS-CoV-2 (**b**) and GX_P2V (**c**) by different doses of breastmilk. The luciferase in the cells was quantified by a microplate luminometer. Cytotoxicity of these drugs to Vero E6 cells was measured by CellTiter-Blue assay. The left and right Y-axis of the graphs represent the mean percentage of inhibition of virus yield and cytotoxicity of the samples, respectively. **d**, **e** RT-qPCR (**d**) and western blot (**e**) analysis of the GX_P2V RNA level in the supernatant treated with different doses of breastmilk (0, 0.16, 0.8, or 4 mg/ml). **f** Plaque assay analysis of the virus titers of the supernatant of control and different concentrations of human breastmilk treatment. **g** Inhibition analysis of whey protein to SARS-CoV-2 pseudovirus by inactivating the protein with heat (90 °C for 10 min) or protease K digestion. **h** Inhibition analysis of commercial bovine formula milk at different concentrations on SARS-CoV-2 pseudovirus infection. **i** Inhibition analysis of whey protein (1 mg/ml) from human, cow, or goat on SARS-CoV-2 and GX_P2V infection. **j** Statistical analysis of recombinant lactoferrin (rLF), bovine lactoferrin (bLF), and human lactoferrin (hLF) on SARS-CoV-2 and GX_P2V infection in Vero E6 cells. **k** Statistical analysis of the IgA antibody effect on SARS-CoV-2 pseudovirus and GX_P2V infection by treating with different doses of anti-IgA antibody. **l** Statistical analysis of the breastmilk blocking SARS-CoV-2 pseudovirus infection through binding the cell surface. **m**, **n** Statistical analysis for breastmilk (4 mg/ml) inhibition of SARS-CoV-2 pseudovirus and GX_P2V attachment (**m**) and entry (**n**). **o** Statistical analysis of breastmilk (4 mg/ml) inhibition of GX_P2V post-entry replication. **p** RNA-dependent RNA polymerase (RdRp) activity analysis for different concentrations of human breastmilk inhibition to SARS-CoV-2’s RdRp activity. MBP, recombinant major basic protein. Values are shown as mean of triplicates ± SD; *p < 0.05, **p < 0.01, ***p < 0.001 by unpaired two-tailed *t* test

We then assessed the impact of human breastmilk on infectious virus production in Vero E6. RT-qPCR analysis of the GX_P2V virus from supernatant showed that even 0.16 mg/ml of breastmilk significantly blocked viral production (Fig. 1d). Western blot of viral nucleoprotein also showed similar results (Fig. 1e). To investigate the infectious virus, we performed plaque assay. As shown in Fig. 1f, the plaque assays showed that live viruses were significantly lower in breastmilk treatment compared to the control group, which confirmed that breastmilk inhibit GX_P2V infection, replication, and production of infectious virions.

Next, to rule out whether protein in breastmilk plays the important role of inhibiting viral infection, we inactivated the protein by applying a temperature of 100 °C for 10 min and protease K digestion, respectively, and tested its role in viral inhibition. As shown in Fig. 1g, breastmilk with treatment by heat and protease K inhibited SARS-CoV-2 pseudovirus infection in both Vero E6 and A549 cells, indicating that the protein in breastmilk plays an important role through its antiviral properties. In addition, SARS-CoV-2 pseudovirus infection could be inhibited by commercial bovine formula milk (EC_50_ = 1.67 mg/ml) in a dose-dependent manner (Fig. 1h).

To investigate whether whey protein from other species could also inhibit SARS-CoV-2 and GX_P2V infection, we chose cow and goat whey protein to analyze their inhibition property. Luciferase assay and RT-qPCR revealed that both cow and goat whey protein inhibited the infectivity of SARS-CoV-2 pseudovirus and GX_P2V, although the inhibition efficiency was relatively lower compared to that of human breastmilk (Fig. 1i). These results indicated that human whey protein has a high concentration of antiviral factors than those from other species.

It was reported that lactoferrin (LF) has broad antiviral effects. Human milk is rich in LF (3–5 g/l in mature milk), which is 10–100 fold higher than that in cow and goat milk.^4^ In addition, LF is the most prominent antimicrobial component in milk.^2^ However, in this study, recombinant lactoferrin, bovine lactoferrin, and human lactoferrin showed limited inhibition of both SARS-CoV-2 and GX_P2V infection at the concentration of 1 mg/ml, suggesting that other components in breastmilk might play important roles in this inhibition (Fig. 1j).

It was also reported that IgA antibody from recovered COVID-19 patients inhibited SARS-CoV-2 infection in vitro.^5^ As the human breastmilk samples were collected before the emergence of COVID-19, the breastmilk donors should have not been infected with SARS-CoV-2. To exclude the impact of IgA- related antibody on viral infection, we utilized the neutralized anti-IgA antibody (1 mg/ml) with different dilutions to mix with the breastmilk (1 mg/ml). As shown in Fig. 1k, the different dilutions of anti-IgA antibody did not influence the inhibitory activity of breastmilk to SARS-CoV-2 and GX_P2V infection, indicating that IgA antibody from breastmilk might not be the key factor inhibiting viral infection and other factors may be responsible for the anti-SARS-CoV-2 activity of whey protein, which merits further study.

To study how human breastmilk inhibits viral infection, we did the viral attachment, entry, and post-entry experiments. As shown in Fig. 1l, human skimmed breastmilk could attach the cell surface to block viral binding and entry. Breastmilk also inhibited viral attachment (Fig. 1m and Supplementary Fig. S3a) and entry (Fig. 1n and Supplementary Fig. S3b) with more than 90% of inhibition rates during both SARS-CoV-2 pseudovirus and GX_P2V infection, suggesting that breastmilk blocks viruses from entry into the cytoplasm. Furthermore, we investigated the impact of human skimmed breastmilk on the affinity between SARS-CoV-2 S protein and ACE-2. As shown in Fig. S3c, the skimmed milk interferes with the affinity between these two proteins in a dose-dependent manner. However, when inactivated by 100 °C for 10 min, the inhibitory activity of viral entry by inactivated breastmilk showed no significant difference compared to the breastmilk without heat treatment. In addition, breastmilk inhibited significantly GX_P2V replication in the present of breastmilk during viral post-entry (Fig. 1o), suggesting that breastmilk inhibits not only viral entry but also viral replication. Furthermore, we also determined whether the possible mechanism of inhibiting SARS-CoV-2 replication is that breastmilk interferes with the RNA-dependent RNA polymerase (RdRp) activity of SARS-CoV-2. Surprisingly, breastmilk could significantly inhibit the RdRp activity of SARS-CoV-2 in a dose-dependent manner (Fig. 1p). These results suggest that breastmilk inhibits both SARS-CoV-2 and GX_P2V infection and replication and shed light on the protection of newborns by breastfeeding from mothers with or without SARS-CoV-2 infection.

Collectively, we reported that human breastmilk inhibits SARS-CoV-2 virus infection in Vero E6 and A549 cell lines. These results suggest whey protein as a direct-acting inhibitor of SARS-CoV-2 and GX_P2V infection and replication by the mechanism of potentially impairing RdRp activity and slightly blocking the affinity of ACE-2 and SARS-CoV-2 S protein. Although our results are limited by the lack of a larger number of breastmilk samples and clinical study, we believe our findings give important clues for the safety of breastmilk feeding and further usage of breastmilk for antiviral drug development, which may be helpful for the development of recommendations on whether mothers with COVID-19 could breastfeed.

## Supplementary information

Supplementary material
