# Supplementary material for: The effect of whey protein on viral infection and replication of SARS-CoV-2 and pangolin coronavirus in vitro
Source: Signal Transduct Target Ther. 2020 Nov 24;5:275. doi: 10.1038/s41392-020-00408-z (PMC7683587; doi:10.1038/s41392-020-00408-z)
Supplement: Supplementary file 1 — Supplementary material [file 41392_2020_408_MOESM1_ESM.docx]

Supplementary Materials for

The effect of whey protein on viral infection and replication of SARS-CoV-2 and pangolin coronavirus in vitro

Huahao Fan^2*^, Bixia Hong^2*^, Yuqian Luo^3*^, Qi Peng^5*^, Liqin Wang^2^, Xiangshu Jin^1^, Yangzhen Chen^2^, Yunjia Hu^2^, Yi Shi^5^, Tong Li^1^, Hui Zhuang^1^, Yi-Hua Zhou^3,4†^, Yigang Tong^2†^, Kuanhui Xiang^1†^

Correspondence to: zgr03summer@126.com (Y.Z.); tong.yigang@gmail.com (Y.T.); Kxiang@bjmu.edu.cn (K.X.)

**This PDF file includes:**

Materials and Methods

Figures. S1 to S3

Table S1

Materials and Methods

**Collecting and handling of milk samples**.

Breastmilk was collected via pumps into sterile containers after disinfecting nipples with 75% ethanol. Samples were frozen in aliquots at -80°C. Skim milk was prepared by centrifuging the samples for 15 min at 4,000 × g at 4°C and the lower aqueous phase was used for further experiments and analysis. Mothers were informed consent. They were not infected with hepatitis B and C viruses and HIV. This study was approved by the ethics committees of the Medical Center.

**Cell lines, coronavirus, and key reagents**

Vero E6 cells (American Type Culture Collection, Manassas, VA, USA) and A549 cells were grown in high-glucose-containing Dulbecco's Modified Eagle Medium supplemented with 10% fetal bovine serum^1^. SARS-CoV-2 pseudovirus was kindly shared by Prof. Youchun Wang (National Institutes for Food and Drug Control, China)^2^. GX_P2V was described recently^3^. Goat and cow whey proteins were purchased from Sigma (USA). The recombined lactoferrin (rLF), human lactoferrin (hLF) and bovine lactoferrin (bLF) were purchased from Sigma (USA).

**Viral infection assay**

SARS-CoV-2 pseudovirus infection was performed as described^2^. The cells were infected with viral inocula of 650 TCID_50_/well. One day post infection (1dpi), the cells were lysed and the luminescence was measured according to the manufacture’s protocol. Cells were infected with GX_P2V at multiplicity of infection (MOI) of 0.01 as described^1^. The messenger RNA (mRNA) levels of GX_P2V and GAPDH were determined by reverse transcription and quantitative real-time polymerase chain reaction (RT-qPCR)^1^.

**Plaque assay for determining virus titer**

The plaque assay for determining virus titer was performed as previously described^1^. Briefly, confluent monolayer Vero E6 cells were infected with GX_P2V with ten-fold dilution from 10^-1^ to 10^-6^. After removing the virus, the cells were washed by PBS and added with 1% agarose overlay to prevent cross-contamination. At 5 dpi, cells were fixed with 4% paraformaldehyde for 1h, followed by staining with Crystal violet for 10 min and washed with water. The plaques were counted and virus titers were calculated.

**Blocking assay**

To study whether the inhibition effects of skimmed breastmilk on SARS-CoV-2 pseudovirus and GX_P2V by binding cell surface to block viral entry, Vero E6 cells were seeded at the 96 well plates with 20,000 cells/well. The skimmed breastmilk at a final concentration of 4mg/ml was added in the wells. After washing away the free breastmilk the next day, the cells were infected with SARS-CoV-2 pseudovirus and cultured for 24h. The luminescence was measured to reflect the viral infection and replication.

**Viral attachment assay**

Vero E6 cells were seeded at the 96 well plates with 20,000 cells/well one day before the infection. Breastmilk at a final concentration of 4mg/ml was mixed with SARS-CoV-2 pseudovirus (650 TCID_50_/well) and GX_P2V (MOI=10) at 4℃ for 1h. The mixture was added into the cells and put at 4℃ for 2h to allow viral attachment to cells. After washing out of free virus, cell surface GX_P2V was extracted and quantified by RT-qPCR^4^. For pseudovirus, the free viruses were washed away and incubated at 37℃ for 24h. The luminescence was measured to reflect the viral infection and replication.

**Viral entry assay**

Breastmilk at a final concentration of 4mg/ml was mixed with SARS-CoV-2 pseudovirus (650 TCID_50_/well) and GX_P2V (MOI=10) at 4℃ for 1h. Vero E6 cells were exposed to the mixture at 37℃ for 1h to allow viral internalization into cells. GX_P2V mRNA was measured by RT-qPCR. For pseudovirus, the free viruses were washed away and the cells were incubated at 37℃ for 24h. The luminescence was measured.

**Viral post-entry assay**

Vero E6 cells were infected with SARS-CoV-2 pseudovirus (650 TCID_50_/well) and GX_P2V (MOI=0.01) and incubated at 37℃ for 1h, respectively. After washing out of the free viruses, the cells were cultured in the media containing breastmilk at a final concentration of 4mg/ml for 24h (SARS-CoV-2 pseudovirus) and 72h (GX_P2V), respectively. Intracellular SARS-CoV-2 pseudovirus was measured by luminescence and GX_P2V was measured by RT-qPCR.

**Viral RNA extraction and quantification**

The RT-qPCR for GX_P2V RNA quantification was performed as previously described^1^. Briefly, total RNA was extracted by the AxyPrep™ multisource total RNA Miniprep kit (Axygene, USA). First strand complementary DNA (cDNA) was synthesized by a Hifair II 1st Strand cDNA synthesis kit with gDNA digester (Yeasen Biotech, China) and quantified by Hieff qPCR SYBR Green Master Mix (Yeasen Biotech, China). The primer sequences were listed in **Supplementary Table 1**. The RT-qPCR amplification of the Taqman method was performed as follows: 50°C for 2 min, 95°C for 10 min followed by 40 cycles consisting of 95°C for 10 s, 60°C for 1 min.

**Western blotting**

Western blotting was performed as described previously^5^. Briefly, the samples were loaded on a 12% SDS-PAGE gel and transferred to a polyvinylidene fluoride membrane. Antibody against nucleocapsid protein of anti-SARS-CoV-2 N protein (Genscript, USA) and GAPDH of anti-GAPDH (Proteintech, USA) were used at 1:3000 dilutions. The second antibody of HRP-conjugated affinipure Goat anti-mouse IgG (H+L) were diluted at 1:20000. SuperSignal® West Femto Maximum Sensitivity Chemiluminescent Substrate (Thermo Scientific, USA) was used for imaging.

**In vitro RdRp activity assay**

The in vitro RNA dependent RNA polymerase (RdRp) activity of SARS-CoV-2 were performed as described previously^6^. Briefly, 1uM of nsp12-nsp7-nsp8 were mixed with 1uM annealed RNA and 0.5 mM NTP in a reaction buffer containing 10 mM Tris-HCl (pH 8.0), 10 mM KCl, 1 mM betamercaptoethanol and 2 mM MgCl2 (freshly added prior usage). Then, the mixture was incubated at 30 °C for 30 min. The annealed RNA was mixed by a 40-nt template RNA (5’-CUAUCCCCAUGUGAUUUUAAUAGCUUCUUAGGAGAAUGAC-3’, Takara) corresponding to the 3’ end of the SARS-CoV2 genome and a 5’-fluorescein label (5’FAM- GUCAUU CUCCUAAGAAGCUA-3’, Takara). The products were denatured by boiling (100℃, 10 min) in the presence of formamide and separated by 20% PAGE containing 9 M urea run with 0.5 3 TBE buffer. Images were taken using a Vilber Fusion system.

**Affinity assay between ACE2 and SARS-CoV-2 Spike protein**

The influence of human breastmilk on the affinity between ACE2 and SARS-CoV-2 was performed as previously described^7^. Briefly, the ACE-2 (Novoprotein, China) was immobilized on the MaxiSORP ELISA plate at 100ng per well in 50 μl of 100 mM carbonate-bicarbonate coating buffer over night at 4 ℃. HRP-conjugated SARS-CoV-2 RBD (Novoprotein, China) at final concentration of 1 ng/μl was mixed with serially diluted human breastmilk. The inactivated breastmilk was performed by heating with 100 ℃ for 10 min. The mixture (100 μl) was added into the ACE-2-coated plate for 1h at room temperature. The absorbance reading at 450 nm and 570 nm were acquired using the Cytation 5 microplate reader (Bio Tek). Inhibition (%) = (1-sample/negative control) X 100%.

**Statistical analysis**

Statistical analyses were analyzed using GraphPad Prism 8 software (GraphPad Software Inc., San Diego, CA, USA). Values are shown as mean of triplicates. Comparisons between the two groups were analyzed using the Student's t tests. Values of *p*<0.05 was considered statistically significant.

**References**

1 Fan, H. H. *et al.* Repurposing of clinically approved drugs for treatment of coronavirus disease 2019 in a 2019-novel coronavirus-related coronavirus model. *Chin Med J (Engl)* **133**, 1051-1056 (2020).

2 Nie, J. *et al.* Establishment and validation of a pseudovirus neutralization assay for SARS-CoV-2. *Emerg Microbes Infect* **9**, 680-686 (2020).

3 Lam, T. T. *et al.* Identifying SARS-CoV-2-related coronaviruses in Malayan pangolins. *Nature* **358**, 282-285 (2020).

4 Fan, H. *et al.* Attachment and postattachment receptors important for hepatitis C virus infection and cell-to-cell transmission. *J Virol* **91** (2017).

5 Xiang, K. H. *et al.* Effects of amino acid substitutions in hepatitis B virus surface protein on virion secretion, antigenicity, HBsAg and viral DNA. *J Hepatol* **66**, 288-296 (2017).

6 Peng, Q. *et al.* Structural and biochemical characterization of the nsp12-nsp7-nsp8 core polymerase complex from SARS-CoV-2. *Cell Rep* **31**, 107774 (2020).

7 Tan, C. W. *et al.* A SARS-CoV-2 surrogate virus neutralization test based on antibody-mediated blockage of ACE2-spike protein-protein interaction. *Nat Biotechnol* **38**, 1073-1080 (2020).


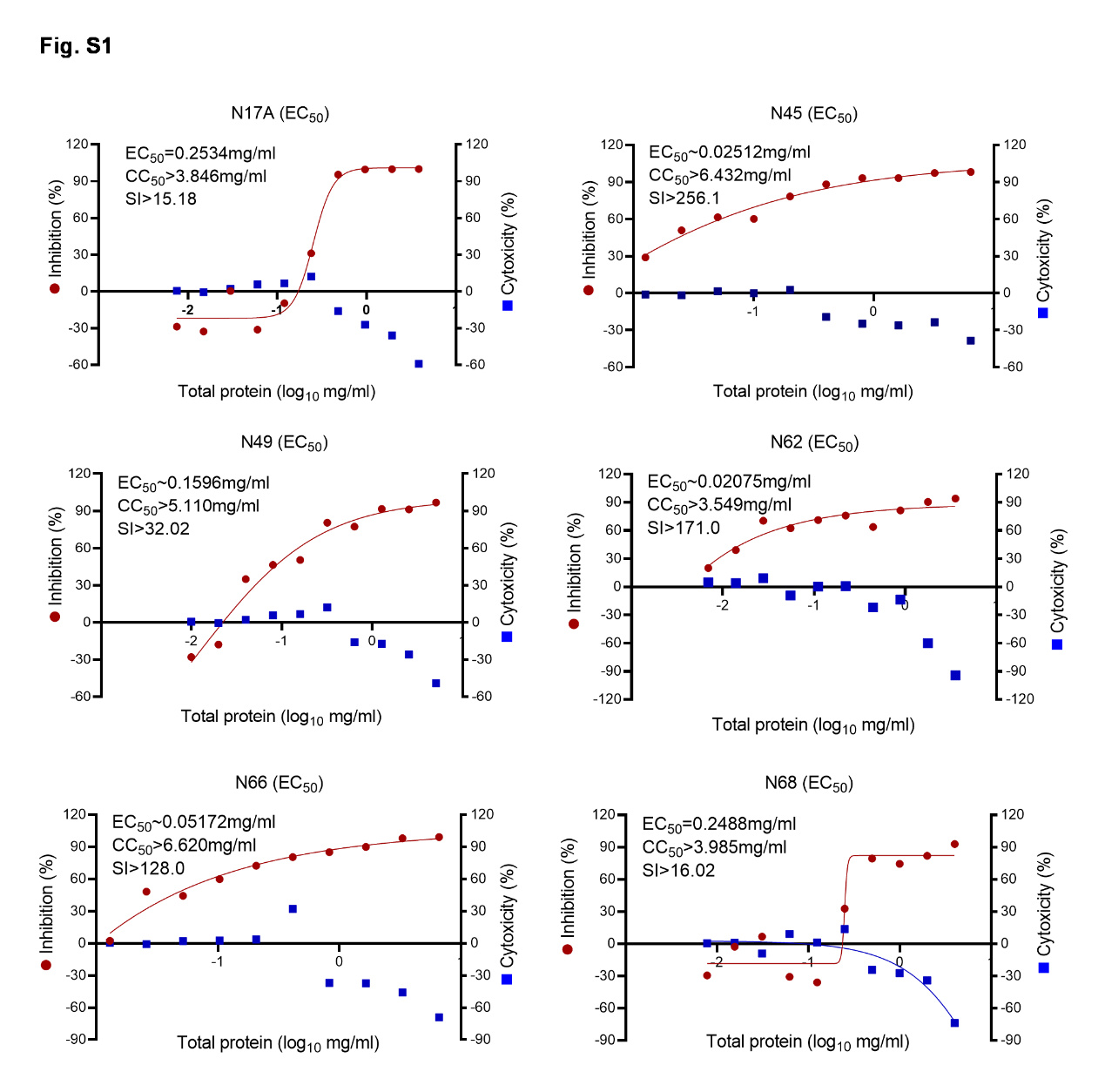


**Fig. S1 The inhibition analysis of SARS-CoV-2 by different doses of breastmilk**. The luciferase in the cells was quantified by the microplate luminometer. Cytotoxicity of these drugs to Vero E6 cells was measured by CellTiter-Blue assay. The left and right Y-axis of the graphs represent mean percentage of inhibition of virus yield and cytotoxicity of the samples, respectively.


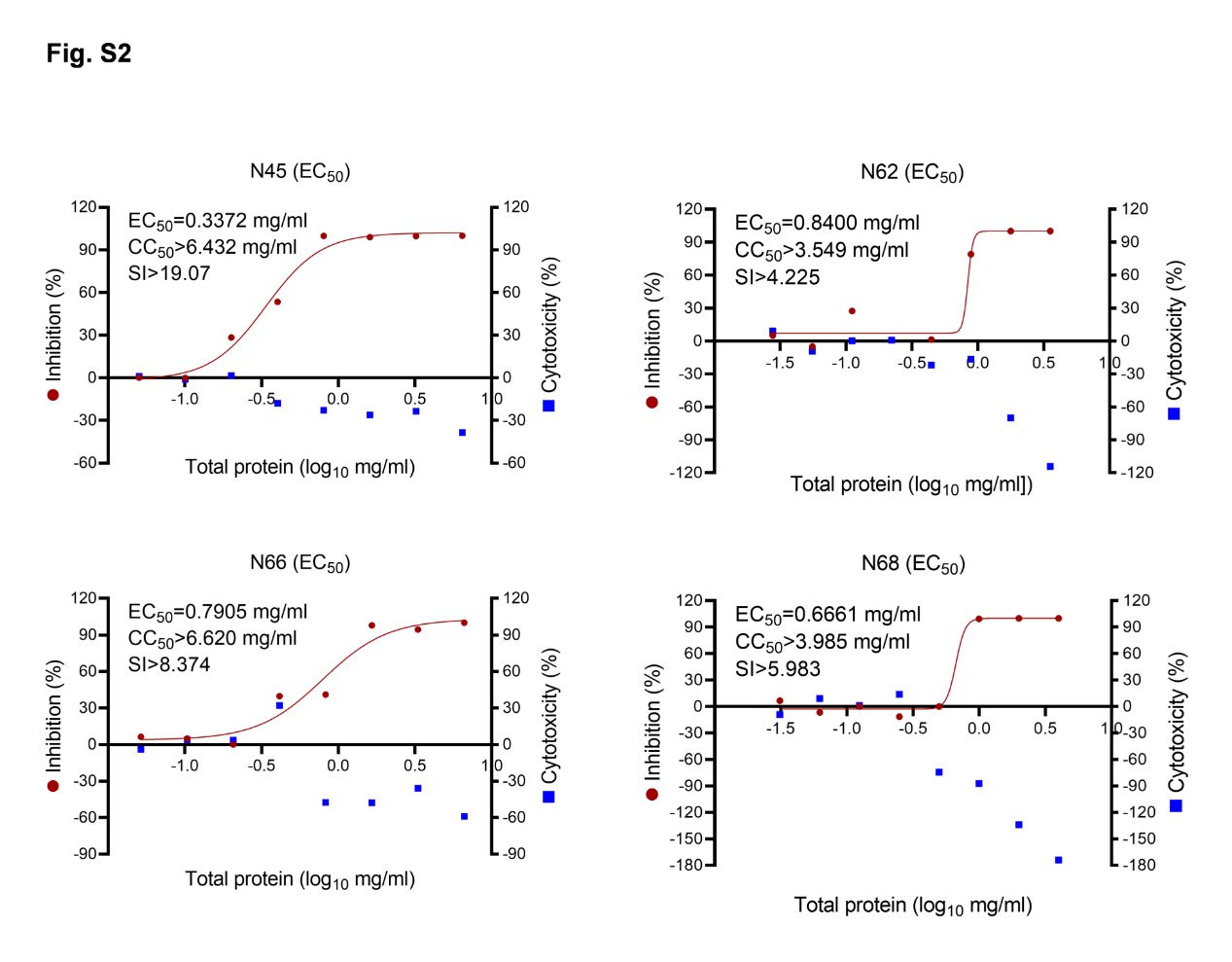


**Fig. S2 The inhibition analysis of GX_P2V by different doses of breastmilk.** The luciferase in the cells was quantified by the microplate luminometer. Cytotoxicity of these drugs to Vero E6 cells was measured by CellTiter-Blue assay. The left and right Y-axis of the graphs represent mean percentage of inhibition of virus yield and cytotoxicity of the samples, respectively.


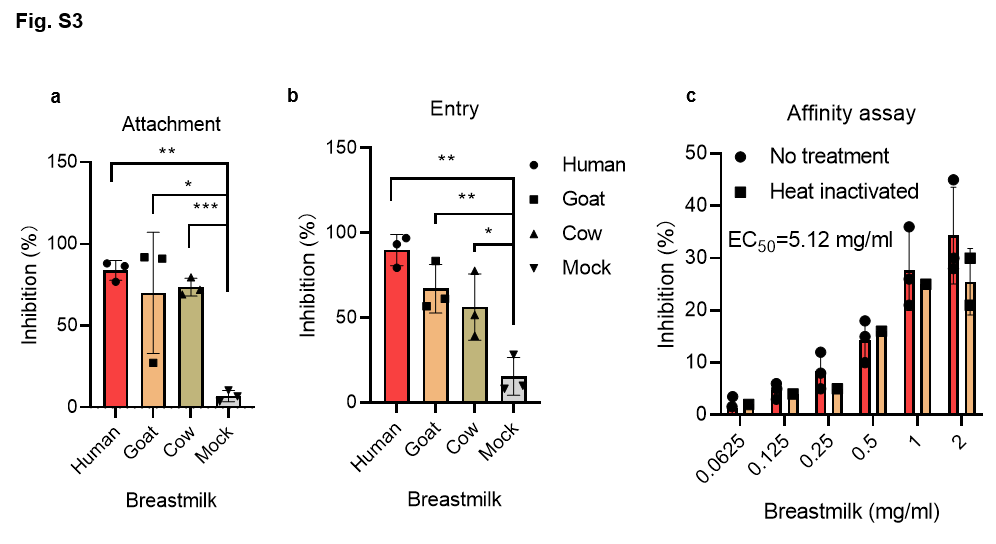


**Fig. S3** **Statistical analysis for breastmilk (4mg/ml) inhibition of SARS-CoV-2 pseudovirus attachment and entry.** Vero E6 infected with SARS-CoV-2 pseudovirus showed that breastmilk inhibited SARS-CoV-2 pseudovirus infection through blocking viral attachment (**a**) and entry (**b**). SARS-CoV-2 pseudovirus infection was measured by luciferase assay. (**c**) Affinity assay for the impact of human breastmilk on the affinity between ACE2 and SARS-CoV-2 RBD. Heat treatment means breastmilk was treated by 100 ℃ for 10 min before added to the affinity testing system. Values are shown as mean of triplicates ± SD, **p*<0.05, ***p*<0.01, ****p*<0.001 by unpaired two-tailed t test.

**Supplementary tables**

**Supplementary Table 1:** **Primers used in the study.**

| **Primer name** | **Sequence** |
| --- | --- |
| CoV-F1 | GGTGATTGCCTTGGTGATATTG |
| CoV-R1 | GCAAGTAGTGCAGAAGTGTATTG |
| CoV-Probe | TCTGTGAGCAAAGGCGGTAGAACC（5’-FAM，3’-TAMRA） |
| GAPDH-q-F | AGCCTCAAGATCATCAGCAATG |
| GAPDH-q-R | ATGGACTGTGGTCATGAGTCCTT |
| GAPDH-q-probe | CCAACTGCTTAGCACCCCTGGCC（5’-FAM，3’-TRAMA） |
